# Sustainable Production of Arecanut Husk Ash as Potential Silica Replacement for Synthesis of Silicate-Based Glass-Ceramics Materials

**DOI:** 10.3390/ma14051141

**Published:** 2021-02-28

**Authors:** Muhammad Fahmi Anuar, Yap Wing Fen, Muhammad Zakwan Azizan, Fida’i Rahmat, Mohd Hafiz Mohd Zaid, Rahayu Emilia Mohamed Khaidir, Nur Alia Sheh Omar

**Affiliations:** 1 Department of Physics, Faculty of Science, Universiti Putra Malaysia, UPM, Serdang 43400, Selangor, Malaysia; fahmianuar6323@gmail.com (M.F.A.); zakwanazizan@gmail.com (M.Z.A.); mhmzaid@upm.edu.my (M.H.M.Z.); nuralia.upm@gmail.com (N.A.S.O.); 2 Functional Devices Laboratory, Institute of Advanced Technology, Universiti Putra Malaysia, UPM, Serdang 43400, Selangor, Malaysia; fidai.rahmat95@gmail.com (F.R.); rahayuemilia.upm@gmail.com (R.E.M.K.)

**Keywords:** arecanut ash, ZnO, SiO_2_, zinc silicate, optical, structural, willemite

## Abstract

Arecanut husk (AH) was selected as a material for silica replacement in the synthesis process of glass-ceramics zinc silicate and also the fact that it has no traditional use and often being dumped and results in environmental issues. The process of pyrolysis was carried out at temperature 700 °C and above based on thermogravimetric analysis to produce arecanut husk ash (AHA). The average purity of the silica content in AHA ranged from 29.17% to 45.43%. Furthermore, zinc oxide was introduced to AHA and zinc silicate started to form at sintering temperature 700 °C and showed increased diffraction intensity upon higher sintering temperature of 600 °C to 1000 °C based on X-ray diffraction (XRD) analysis. The grain sizes of the zinc silicate increased from 1011 nm to 3518 nm based on the morphological studies carried out by field emission scanning electron microscopy (FESEM). In addition, the optical band gap of the sample was measured to be in the range from 2.410 eV to 2.697 eV after sintering temperature. From the data, it is believed that a cleaner production of low-cost zinc silicate can be achieved by using arecanut husk and have the potential to be used as phosphors materials.

## 1. Introduction

Arecanut is a well-known tropical crop that belongs to Arecaceae family that are commercially and socially important in south-east Asian countries. India, Sri Lanka, Bangladesh, Malaysia, Indonesia, and Philippines are the main countries that grow arecanut [1]. The arecanut husk (AH) constitutes about 65–80% of the total weight and volume of the fruit. Generally, the arecanut is used for chewing in some Asian countries and the husk of the arecanut fruits is removed due to it has no traditional use. Occasionally, AH is being used as fuel for the processing of the nut. As a result, large heaps of the AH biomass caused environmental issues due to the lack of proper disposal techniques for the waste husks. Hence, the rising issues due to the growth of arecanut industry have inspired researchers to find alternative uses for the waste AH.

Several studies had been conducted to utilize this renewable biomass for different applications. The arecanut husk has been used as a supportive cementitious material [2], adsorption of malachite green from aqueous solutions [3], ethanol production [4], as natural reinforcement in biodegradable polymer composites [5], and was also studied in domestic wastewater treatment [6]. Despite that, there are only a few related researches of AH that used it as a source of silica in glass-ceramics applications.

In recent years, the development of glass-ceramics has attracted a lot of attention from researchers due to its producibility from waste materials. Due to its compositions, AH ash is considered to have the potential in the production of silicate-based glass-ceramics. The major component found in AH ash is silica along with some metallic oxide such as alumina, magnesium oxide, and potassium oxide [2]. In addition to being environmentally friendly, the production of glass-ceramics derived from waste materials is also low-cost and thus reduces the dependency on natural resources in glass-ceramics production.

Zinc silicate is also known as willemite is a glass-ceramics material that has been identified as a capable host matrix for doping with various transition metals or rare-earth ions for their luminescence properties [7,8]. It has been used widely as a phosphor in fluorescent lamps, television, display, and lighting devices [9]. Due to its great luminescence properties, researchers are more focused to develop a new method to synthesis zinc silicate-based glass-ceramics for potential phosphors materials.

In the present work, the preparation of zinc silicate from waste AH has been described and the sintering effects on structural and optical properties of the glass-ceramics materials are also investigated.

## 2. Experimental Procedure

### 2.1. Preparation of Arecanut

**Figure 1:**
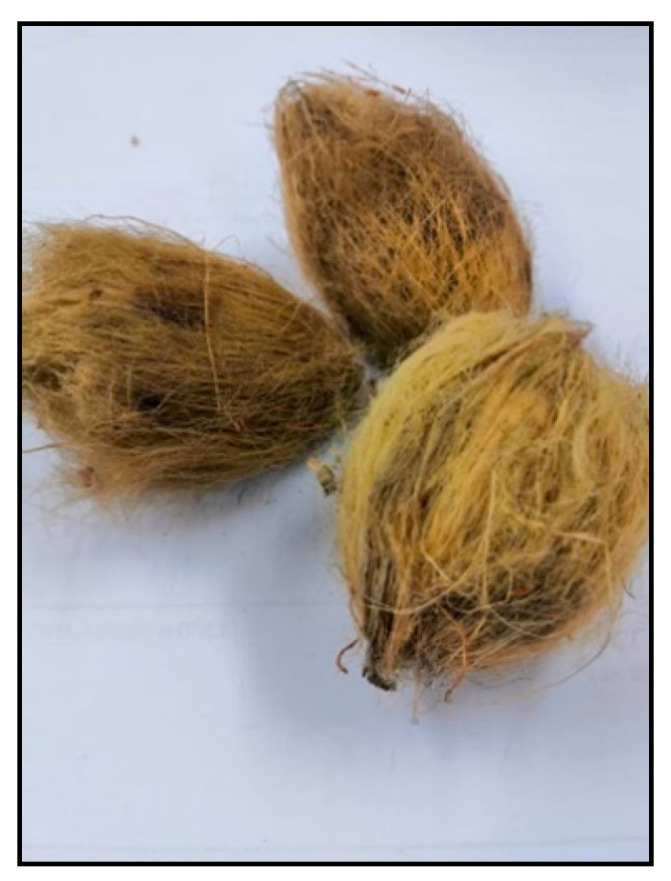
Cleaned arecanut fruit collected from a local farm.

In this experiment, arecanut husks were subjected to pyrolysis. Arecanut waste (Figure 1) was collected from a local farm in Selangor, Malaysia. The husk was cleaned by distilled water and dried up in the oven at 70 °C for 24 h. This precaution step was carried out to avoid any contamination, removed the unwanted residues, and kill all the insects that have been living in the husk. After that, the husk was burned in the electrical furnace at 700 °C, 800 °C, and 900 °C to determine the optimum temperature needed to extract the highest percentage of SiO_2_ from arecanut husk. Furthermore, the powder was then sieved into fine powder of approximately 45 μm.

### 2.2. Synthesis of Zinc Silicate

To prepare Zn_2_SiO_4_ based arecanut waste silica, 10 g batch of the mixture first weighed using digital weighing machine. The mixture powder then thoroughly mixed via milling process using a ball milling jar for 4 h. After the milling process, the mixture was melted in an alumina crucible at 1450 °C in the air with a heating rate of 10 °C/min for 2 h in an electrically heated furnace. The molten mixture was immediately poured into water to obtain a transparent glass fritz. The glass fritz was naturally dried at room temperature for 1 day and later crushed and sieved into fine powder of about 45 μm. Then, the sample was sintered at 600–1000 °C for further characterization. 

### 2.3. Characterization

**Figure 2:**
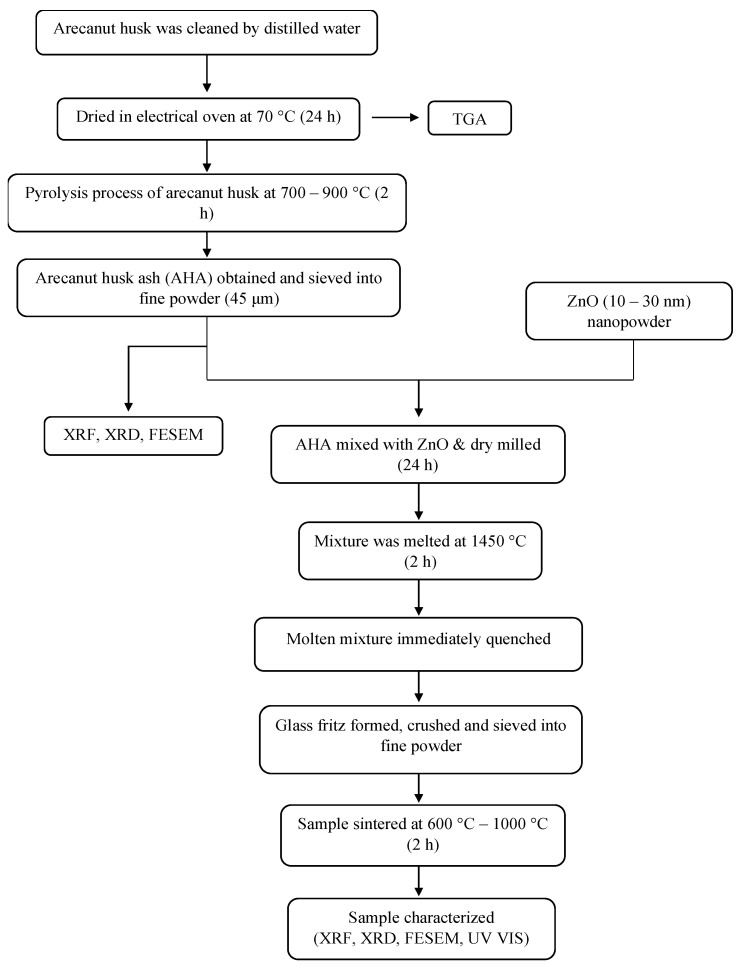
The methodology of producing zinc silicate from waste arecanut husk.

In this work, thermogravimetry analysis (TGA) was carried out by using a Mettler Toledo TGA/DSC 1HT (Mettler Toledo, Küsnacht, Switzerland) to determine the thermal stability of the arecanut husk in the presence of oxygen gas. Quantitative measurement of the sample’s elemental composition was carried out using X-ray fluorescence (XRF) XRF-EDX-720/7000 spectroscopy (Shimadzu, Kyoto, Japan). Furthermore, the phase identification of the sample was analysed by using X-ray diffraction (XRD) Phillips X’Pert High Pro PANanalytical Diffractometer (Malvern PanAnalytical, Almelo (Netherland), and Malvern (UK)). The morphological structure of the sample was observed under the field emission scanning electron microscopy (FESEM), Nova NanoSEM 30 (FEI, Hillsboro, OR, USA) under 25,000× magnification level. In addition, the absorbance properties of the sample were analysed by UV-3600 Shimadzu (Shimadzu, Kyoto, Japan). The overall methodology of the experiment is illustrated in Figure 2.

## 3. Results and Discussion

### 3.1. Arecanut Husk (AH)

**Figure 3:**
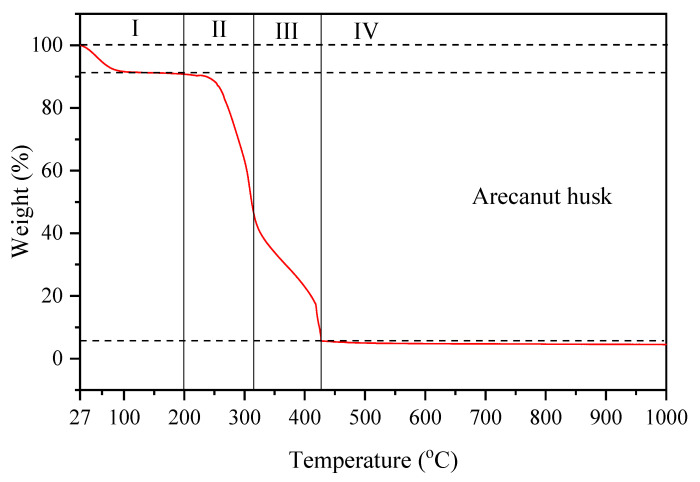
Thermogravimetry analysis (TGA) of arecanut husk.

Thermal analysis of the arecanut husk was conducted by using thermogravimetry analysis (TGA) at a temperature range of 27–1000 °C. A graph of temperature against weight was plotted as shown in Figure 3. The percentage of weight loss at each stage can be determined from the plotted graph. The initial stage (I) of the weight loss corresponds to the release of moisture from the arecanut husk. At this stage, about 8.97% of the total weight of arecanut husk decomposed due to loss of water content and light volatile compounds [10,11,12]. Afterward, the second stage (II) of weight loss occurred in the range of 200 °C–315 °C. Above 200 °C, the thermal resistance of agriculture waste gradually [13]. About 44.50% of the weight of AH was loss at this stage due to the decomposition of hemicellulose [14]. Stage (III) occurred at temperature between 315 and 430 °C and the arecanut husk undergone about 40.77% weight loss, which is associated with the decomposition of cellulose. Stage (II) and (III) of the TGA graph undergone rapid weight loss was due to the devolatilization of organic compounds of hemicellulose and cellulose [11,15]. Decomposition of hemicellulose occurred at a lower temperature (180–340 °C) and cellulose (230–450 °C) [14]. At this stage (IV), AH produces about 5.76% of ash after the pyrolysis process. The stabilization of mass loss for AH at stage (IV) specifies the complete burning process of volatile components and proposes a temperature above 600 °C for the pyrolysis process for char formations. To sum up, the TGA was carried out and the weight degradation analysis of the AH was discussed and deemed suitable to be used in this work.

### 3.2. Arecanut Husk Ash

The elemental analysis of arecanut husk ash (AHA) pyrolyzed at different temperature was determined with XRF as in Table 1. Arecanut ash sample was pyrolysed at three different temperatures for 2 h to determine the optimum silica composition in the ash. From the table, it is obvious that predominant SiO_2_ elements was detected at temperature range from 700 to 900 °C. Other elements such as Al_2_O_3_, SiO_2_, P_2_O_5_, Cl, K_2_O, CaO, Fe_2_O_3_, and ZnO were also detected in the AHA as the minority elements. AH that was pyrolyzed at 900 °C shows the highest percentage of SiO_2_ which is 45.43% and thus suitable for further synthesis of zinc silicate based glass-ceramics. 

**Figure 4:**
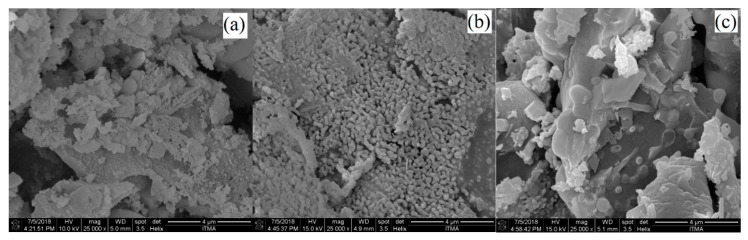
FESEM images of arecanut ash burned at (**a**) 700 °C (**b**) 800 °C (**c**) 900 °C under magnification of 25,000×.

Figure 4 illustrates the FESEM images for arecanut ash burned at different temperatures, which are 700 to 900 °C under 25,000× magnification. As shown in the figures, the results presented that all the samples have irregular medium-sized particles distribution with crushed shape structure. FESEM observation of AHA showed aggregation and irregularity in shape and size [16], which is different in comparison with coconut husk ash (CHA) that showed rod-like structure [17].

### 3.3. Zinc Silicate

**Figure 5:**
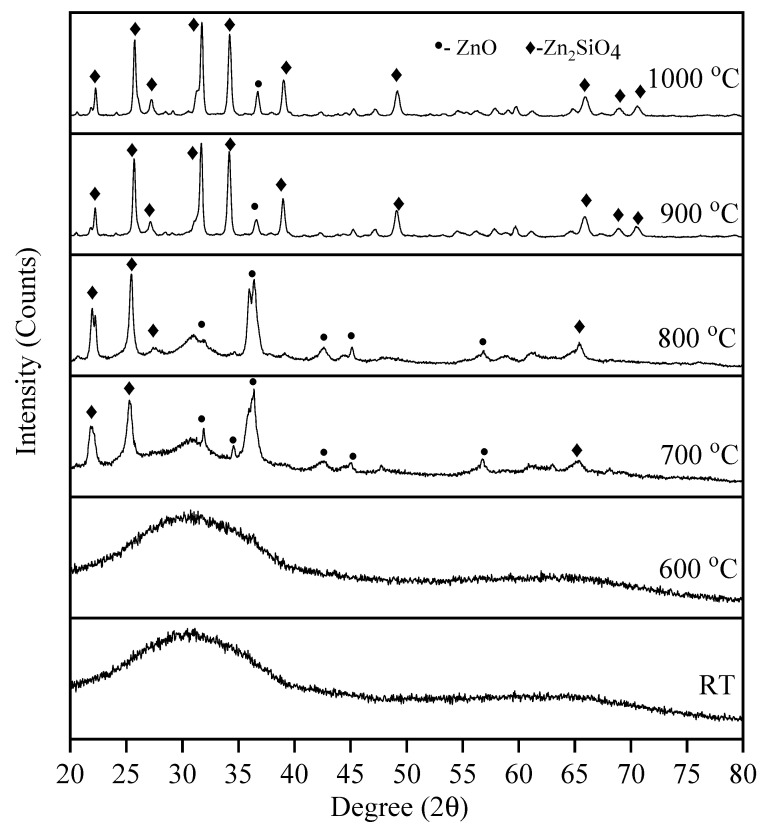
XRD patterns of Zn_2_SiO_4_ sintered at different temperatures.

The crystalline or amorphous nature of the samples was analysed using XRD. Figure 5 consists of XRD patterns of the sample sintered at various sintering temperatures from 600 °C to 1000 °C for 4 h. The unsintered glass sample (RT) was presented with no diffraction peaks, which indicate the amorphous nature of the sample. Besides, at 600 °C, the sample was still in amorphous structure due to the low sintering temperature that does not induced the crystal growth of the sample. The crystalline process begins at temperatures higher than 700 °C. It was noticed that the characteristics peak of the sample at this temperature shows the lowest intensity that indicated the sample has poor crystallinity. Three major peaks appeared indicating the presence of Zn_2_SiO_4_ and according to Inorganic Structural Database (ICSD) no. 00-037-1485, the Zn_2_SiO_4_ has an orthorhombic crystal structure same reported in previous research [18,19,20,21]. Besides that, diffraction pattern that resembled ZnO (ICSD 00-036-1451) was also found at this temperature.

At 800 °C, more elements of Zn_2_SiO_4_ started to appear at 27.30°. Moreover, as the temperature increased to 900 °C, strong and high-intensity peaks of rhombohedral Zn_2_SiO_4_ appeared and fewer ZnO peaks were observed [22]. The formation of the Zn_2_SiO_4_ has the highest peak at this temperature compared to other sintering temperatures. It was explained that ZnO is the dominant diffusing species in ZnO/Al_2_O_3_ couple at higher temperatures greater than 700 °C [23] and it was also believed that the same result can be obtained in the case of ZnO/SiO_2_ due to the similarities between SiO_2_ and Al_2_O_3_ such as amorphous nature and large optical bandgap [24]. For the sample sintered at 1000 °C, the zinc silicate phase have the highest peak intensity in comparison to other temperatures. At this temperature, peaks identified as ZnO still exists at 36.75°. This phenomenon indicated that at 1000 °C sintering temperature, the formation of Zn_2_SiO_4_ was higher due to the increase in crystal growth, and the peaks become more intensified due to the increasing crystallinity of the sample [25].

**Figure 6:**
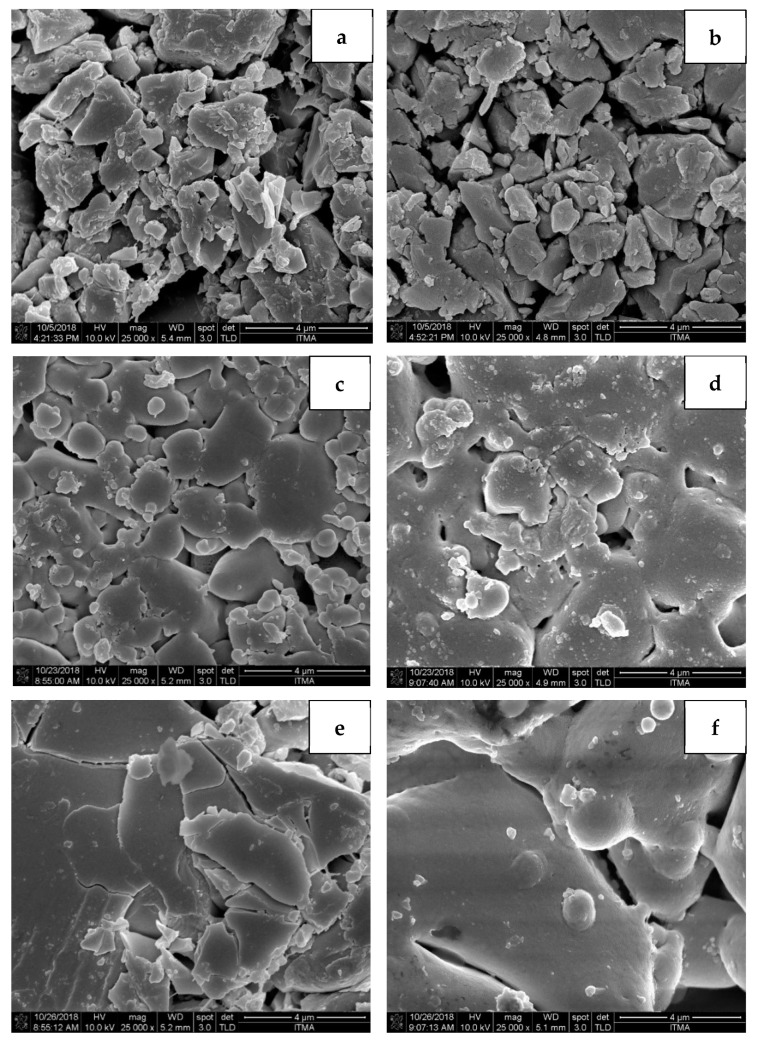
The microstructure images of zinc silicate sintered at different sintering temperature of (**a**) RT, (**b**) 600, (**c**) 700, (**d**) 800, (**e**) 900, and (**f**) 1000 °C.

**Figure 7:**
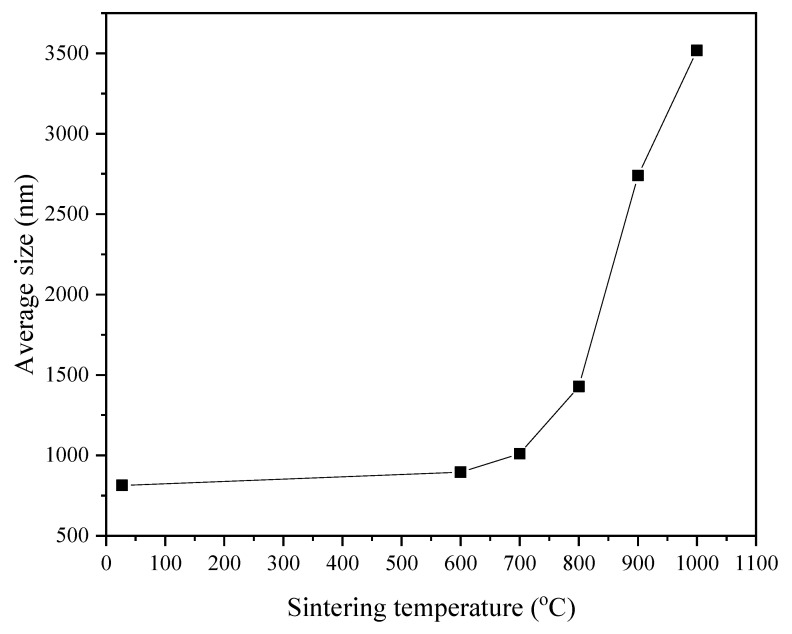
The average grain size of zinc silicate sintered at different sintering temperatures of RT, 600, 700, 800, 900, and 1000 °C.

FESEM analysis was performed to study the shape, size, and morphology of the Zn_2_SiO_4_ samples. FESEM microstructures of the sintered samples are presented in Figure 6a–f under magnification of 25,000×. From the FESEM micrographs, it was observed that the prepared sample was not uniform and consisted of irregular particle size distribution with the average grain sizes of 813 nm. After sintered at 600 °C, the sample was observed to gain in sizes with the average grain sizes measured to be 895 nm. When the sintered temperature increased to 700–1000 °C, the morphology of the Zn_2_SiO_4_ glass-ceramic became granular and show more homogeneous distribution [26]. Furthermore, samples sintered at 700 °C to 1000 °C showed larger grain sizes with an increase in sintering temperature measured from 1011 to 3518 m. Thus, it is confirmed that the grain size of the samples showed increasing trends as the sintering temperatures increased due to the increase in its growth rate and crystallinity [27,28]. The relations between sintering temperature and the average grain sizes of the samples are illustrated in Figure 7.

**Figure 8:**
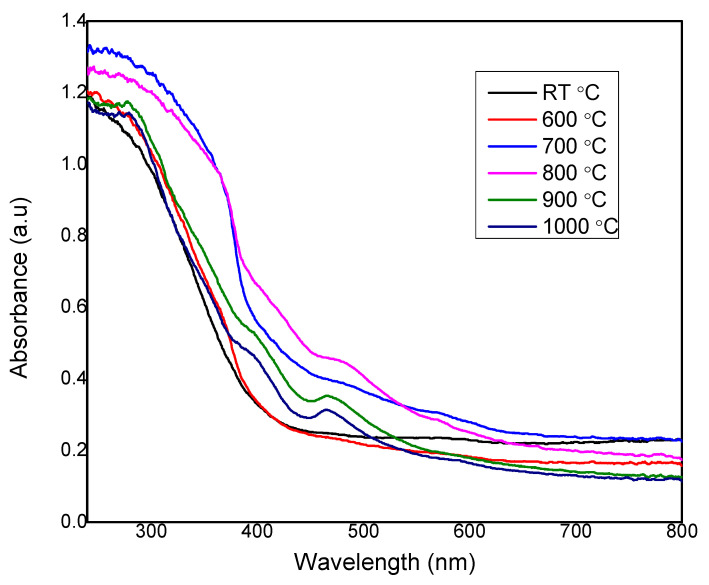
The absorbance spectrum of zinc silicate sintered at different sintering temperatures of RT, 600, 700, 800, 900, and 1000 °C.

UV-Visible spectroscopy measurement for the glass-ceramics samples was performed at room temperature in spectral regions between 220 and 800 nm. The absorption spectra of Zn_2_SiO_4_ sintered at different sintering temperatures are shown in Figure 8. According to the result presented, the curve showed that intensive absorption occurred in the range of 200–300 nm due to the scattering by Zn_2_SiO_4_ nanocrystal similar as reported by [29]. It indicates that the highest absorbance intensity occurs when the sintering temperature was at 700 °C and 800 °C, meanwhile at 1000 °C, the absorption intensities were low due to the formation of pure willemite phase, which distinguishes the ZnO hexagonal structure [24]. Based on XRD data, ZnO hexagonal phase formed when the sample was heated at 700 °C and 800 °C due to the fact that the absorbance of the sample at 700 °C and 800 °C were the highest compared to other temperatures. At higher temperature of 900 °C to 1000 °C, the absorbance decreases due to the increase in the willemite phase intensity [30]. Lastly, the absorbance of unsintered temperature and 600 °C sintering temperature has low intensity because the samples still in amorphous state.

To determine the optical band gap of various samples were different, the graph of (*αhv*)*^n^* versus *hv* was plotted. The relation between the absorption coefficient and photon energy can be related by using Beer–Lambert Law [31] as in Equation (1).

(1) $$\alpha =\frac{k{\left[hv-E_{\mathrm{gap}}\right]}^{n}}{hv}$$

Equation (1) can be rearranged into:

(2) $${\left(\alpha hv\right)}^{1/n}=hv-E_{\mathrm{gap}}$$

where α is the absorbance coefficient, *h* is plank constant, *v* is the frequency, *n* is the transition, and *E*_gap_ is the optical band gap [32]. In this work, *n* = ½ was inserted into Equation (2) to form Equation (3)

(3) $${\left(\alpha hv\right)}^{2}=hv-E_{\mathrm{gap}}$$

**Figure 9:**
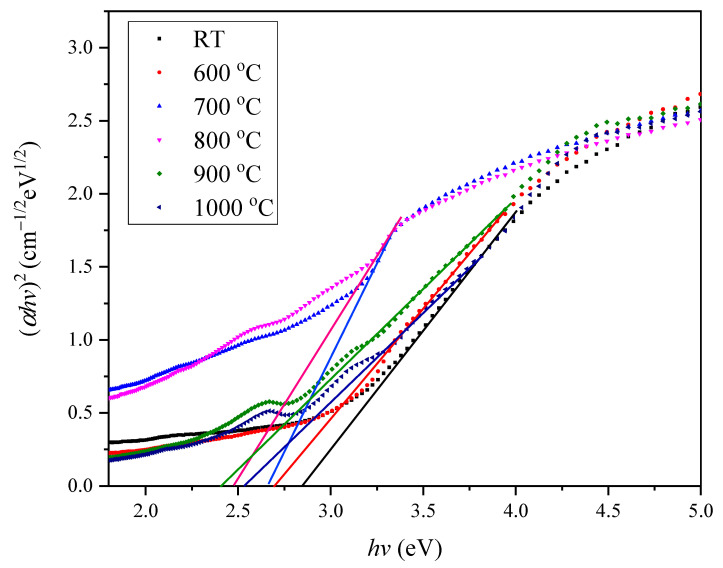
The optical band gap of zinc silicate sintered at different sintering temperatures of RT, 600, 700, 800, 900, and 1000 °C.

From the graph, each best fit line was drawn until it reaches the x­axis to determine the optical band gap. To investigate the energy band gap for each sample, a graph was plotted for each one of different sintering temperatures. Figure 9 shows the graph of energy band gap for the direct transition. Referring to that figure, the energy band gap of unsintered Zn_2_SiO_4_ was 2.847 eV. In the visible light region, the zinc silicate displayed higher peaks of absorption due to Van Hove singularities. Different sintering temperatures caused the zinc silicate experienced significant changes to the Van Hove peaks. Next, the energy band gap for the temperature at 600 °C and 700 °C of the sample were 2.697 eV and 2.660 eV, respectively. The energy band gap dropped slightly at temperature 800 °C (2.474 eV) and 900 °C (2.410 eV) but it increased at 1000 °C, which was 2.530 eV. In comparison, the band gap value of 2.970 to 4.540 eV [8,17,33,34,35] was obtained for zinc silicate from previous research that indicated the zinc silicate has flexible band gap value (optical properties) that were influenced by different steps in the synthesis process.

Overall, the decreasing trend of the optical band gap was observed for both graphs as the temperature keeps increasing to 900 °C but increased after being heated at 1000 °C. The increase in the energy band gap was due to the improvement of the sample crystallinity and quantum size effect [36]. The energy band gap tends to decrease as the temperature increased due to band gap narrowing and smaller separation distance of an electron-hole [37].

Altogether, research data were analysed and discussed with detail. Without a doubt, AH was indeed a suitable material as a substitute material for silica. Both structural and optical properties of the synthesized zinc silicate from the AH exhibit good properties to be used in optical applications especially as phosphors material.

## 4. Conclusions

In this investigation, the arecanut husk (AH) was explored as it has great potential to be used as silica replacement. It is estimated that the average purity of SiO_2_ in AHA ranged from 29.17 to 45.43% with irregular shape and size distribution. Furthermore, zinc silicate was successfully prepared by adding AHA and ZnO and shows high diffraction intensity that indicates the sample have high crystallinity at higher temperature. After the sintering process, the sample was measured to have increased in sizes up to 3517.8 ± 3.08 nm at 1000 °C. In addition, the optical band gap of the sample decreased upon higher temperature but at 1000 °C the band gap increased to 2.530 eV due to improve in crystallinity. Altogether, this works emphasis the potential of AH as a silica replacement and as well as the possibility to be used as a phosphor material.

## Figures and Tables

**Table 1:** Elemental composition of arecanut husk ash (AHA) at different sintering temperature.

Elements	Percentages of Compositions (%)		
	700 °C	800 °C	900 °C
Al_2_O_3_	0.64	2.00	0.00
SiO_2_	29.17	44.14	45.43
P_2_O_5_	26.91	14.64	18.62
Cl	0.75	0.33	0.30
K_2_O	16.65	7.28	10.35
CaO	20.07	13.99	14.16
Fe_2_O_3_	4.02	13.57	9.62
ZnO	0.57	0.66	0.54

## Data Availability

The data presented in this study are available on request from the corresponding author.
